# Herniation of the uterus, ovaries and fallopian tubes into the canal of Nuck in a 4-month-old child: A rare entity

**DOI:** 10.4102/sajr.v24i1.1935

**Published:** 2020-11-04

**Authors:** Dharmendra Kumar, Saurabh Maheshwari, Uddandam Rajesh, Darshan Grewal, Vibhuti Maria

**Affiliations:** 1 Department of Radiodiagnosis and Imaging, Armed Forces Medical College, Pune, India

**Keywords:** Canal of Nuck, Canal of Nuck hernia, Uterine herniation, Pediatric hernia, Uterine herniation, Ovarian herniation

## Abstract

Partial or complete failure of obliteration of the processus vaginalis in the female results in the formation of a potential space known as the canal of Nuck, into which various organs and/or collections can herniate. A 4-month-old female presented with a left labial mass related to herniation of the uterus, ovaries and fallopian tubes through the canal of Nuck. Early diagnosis is important as there is a high risk of ovarian torsion and incarceration.

## Introduction

The canal of Nuck is a potential space because of patency of the processus vaginalis in females. This was first described by the Dutch anatomist, Anton Nuck, in 1691.^1^ The processus vaginalis is a tubular fold of parietal peritoneum, which is seen in both sexes during foetal development. It is seen after 12 weeks of gestation and usually obliterates in early life. This obliteration occurs gradually from a superior to an inferior direction.^2^ The patency of the processus vaginalis in females leads to the presence of a potential space between the peritoneum, inguinal canal and labia majora, which is known as the canal of Nuck. Its distal part remains patent in males and forms the tunica vaginalis of the testis.

The gubernaculum is another structure that is closely associated with the processus vaginalis and lies anterior to it in the inguinal canal. In females, it attaches to the midpoint of the uterus and prevents the descent of the ovary into the inguinal canal.^3^ It also assists in maintaining the normal anteverted and anteflexed position of the uterus.^2^ Its adult homologues are the round ligament and the ovarian ligament. The gubernaculum plays a key role in testicular descent in males.

There may be herniation of various organs and/or collections through the canal of Nuck. The contents of the hernial sac may include omental fat, bowel loops, ovary, fluid, fallopian tube and urinary bladder. In extremely rare cases, the uterus can also herniate. It usually presents as a groin swelling or a labial mass, which may or may not be associated with pain.^3^

Here, we report a case study of a female infant, who presented with a left labial swelling and was found to have herniation of the uterus and bilateral adnexa into the canal of Nuck.

## Patient presentation

A 4-month-old female infant presented with a left labial swelling that increased in size upon crying. The swelling was gradually increasing in size and was noticed by her parents whilst bathing her. They brought the child to the hospital 2 weeks after they first noticed the swelling. The infant was born by natural vaginal delivery at 34 weeks of gestation and had a birth weight of 1.6 kilograms (kg) with an uneventful post-natal course.

**FIGURE 1:**
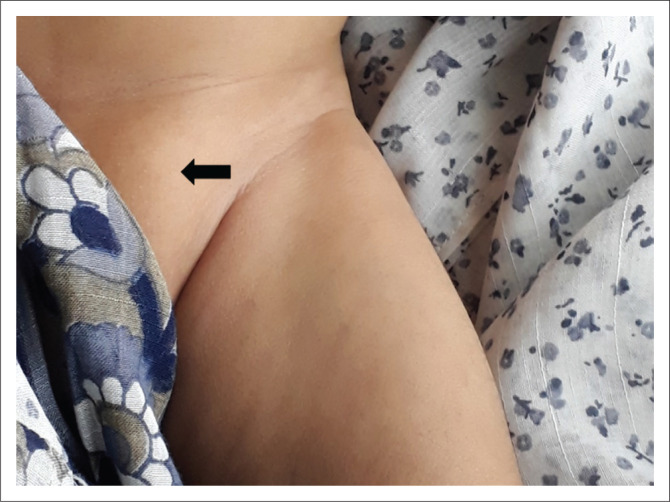
Frontal photograph of the pelvic region of the patient shows the swelling in the left inguinal region (solid arrow).

On physical examination, a soft lump was seen in the left inguinal region with extension to the left labia majora (Figure 1). The lump was non-tender and the overlying skin was normal. It was reducible on manual compression but recurred upon crying. The infant was referred for a sonographic examination to assess the cause and contents of the left inguinal mass.

**FIGURE 2:**
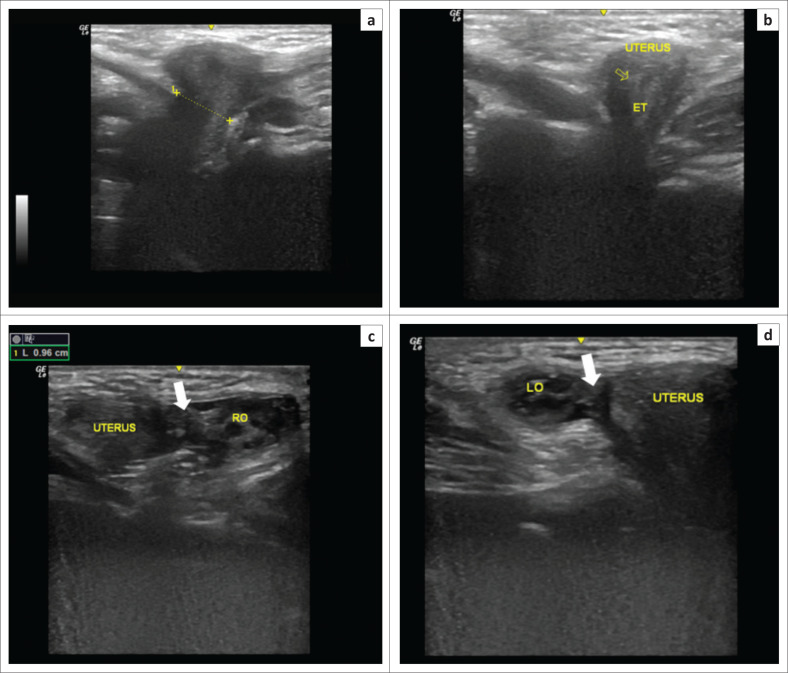
Ultrasound images of the left inguinal region (a to d) show a 9.6 mm size defect (dotted line in [a]) with the uterus herniating through it (void yellow arrow in[b]). The endometrial cavity (ET) can be seen within the uterus (b). Figure c and Figure d demonstrate the herniated right (RO) and left ovaries (LO) along with fallopian tubes (white solid arrows).

She was examined with a NextGen Logiq E portable ultrasound (GE Medical Systems, Milwaukee, Wisconsin, USA) with a 12.5 megahertz (MHz) frequency transducer. The sonographic examination revealed a 9.6 millimetre (mm) defect in the left inguinal fascia with herniation of the pelvic organs through it (Figure 2a and b). Its contents included the uterus, which was identified by the presence of an endometrial lining. Two oval masses with multiple small cystic structures representing ovaries were also seen herniated through this defect (Figure 2c and d). Minimal free fluid was present in the sac. These structures were not seen in the pelvis, which confirmed their herniation into the sac.

**FIGURE 3:**
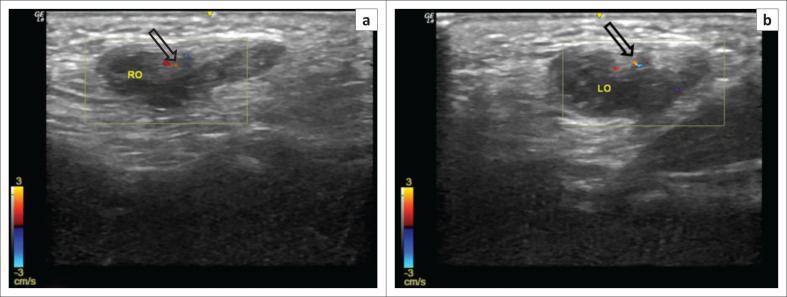
Colour Doppler ultrasound images (a and b) of the herniated contents show preserved vascularity (void black arrow) in the right (a) and left (b) ovaries.

On probe compression, a part of the uterus was seen to reduce into the inguinal canal. Doppler ultrasound demonstrated normal blood flow to all herniated organs (Figure 3). A final diagnosis of a left-sided canal of Nuck hernia containing the uterus, both ovaries and fallopian tubes was made.

## Management and outcome

The patient was managed with an elective repair of the hernia. The ultrasound findings were confirmed intraoperatively. The hernia sac contained the uterus, bilateral ovaries and bilateral fallopian tubes. The broad ligament of the uterus was stretched. The herniated organs were mildly congested without any evidence of ischaemia. They were reduced, the hernial sac was ligated and the deep inguinal ring was closed. The contralateral side was explored and was found to be normal. The surgery and the post-operative course were free of complications.

## Discussion

A palpable mass in the inguinal region of female infants has a relatively narrow range of differentials, including lymphadenopathy, canal of Nuck hernia, hydrocele of canal of Nuck, lipoma, Bartholin gland cyst, lymphatic malformations, haemangiomas, epidermal cysts and rhabdomyosarcoma.

The incidence of paediatric inguinal hernia ranges from 0.8% to 4.4%.^4^ However, they are 6–10 times more common in male infants and are predominantly of the indirect variety.^4^ In females, the hernia through a patent inguinal canal is known as the canal of Nuck hernia. In one study of 22 cases of canal of Nuck hernias, the patient ages ranged from 1 to 137 months, with a mean age of 51 months.^5^ Prematurity is a well-known risk factor for this condition.^6^ Other risk factors include lung disease and mechanical ventilation, where herniation is related to increased intra-abdominal pressure.^7^

Herniation of the uterus through the canal of Nuck is extremely rare. There have been only nine reported cases of herniation of the uterus with only three reported cases of herniation of both adnexa.^8^ Interestingly, all the cases with bilateral herniation have been reported to be on the left side, including our case study. The etiopathogenesis of herniation of the uterus is not clear. Okada et al. suggested multiple hypotheses, including traction induced by broad ligament, weakness of the pelvic suspensory ligaments and crying leading to high intra-abdominal pressures.^9^

Although clinical examination can help in narrowing the differential diagnosis, ultrasound is the primary modality of choice for evaluation of these inguinal masses. Ultrasound is easy and non-invasive, without carrying the risks associated with ionising radiation. It can characterise the mass and provides information regarding its size, shape, location, internal contents and vascularity. It can also provide a dynamic evaluation of the mass in real-time. Magnetic Resonance Imaging (MRI) may be used in difficult cases. However, it has inherent challenges because of the need for sedation or anaesthesia in young patients and the long examination time.

Herniation of the ovary through the canal of Nuck should be diagnosed early, as there is a high risk of ovarian torsion as well as incarceration in these cases, and therefore prompt surgical intervention is necessary.^10^ Various approaches have been described in the literature regarding the surgical management of these cases. These include a simple herniorrhaphy (high ligation of the sac followed by tissue repair),^9^ with or without the closure of the deep inguinal ring.^11^ Some authors have also recommended the anterior repair of the inguinal canal in these cases.^12^ The mesh repair is contraindicated in the paediatric hernia repair because of an increased risk of complications.^13^ There is a high risk of surgical complications in this entity due to the adhesions between the herniated organs and the wall of the hernia sac.^11^

## Conclusion

Hernia of the canal of Nuck is an uncommon entity that should be always be considered in a young female presenting with an inguinal or labial mass. Radiologists should be well aware of this condition and ultrasound is the modality of choice for initial investigation. The knowledge of the anatomy and embryology of the canal of Nuck is essential for interpretation and a correct diagnosis. The present case study illustrates a rare variant of this condition containing the uterus and bilateral adnexa. Early diagnosis of this condition is essential for prompt surgical management.
